# Fossicking for microbial defense system: novel antiviral immunity

**DOI:** 10.1038/s41392-020-00423-0

**Published:** 2020-11-27

**Authors:** Ping Lin, Qun Wu, Min Wu

**Affiliations:** 1 Department of Biomedical Sciences, School of Medicine and Health Sciences, University of North Dakota, Grand Forks, ND 58203 USA; 2 Wound Trauma Medical Center, State Key Laboratory of Trauma, Burns and Combined Injury, Daping Hospital, Army Medical University, Chongqing, 400042 China; 3 Department of Pediatrics, Ruijin Hospital affiliated to Shanghai Jiao Tong University School of Medicine Shanghai, Shanghai, China

**Keywords:** Biophysics, Microbiology, Infection

**Fig. 1:**
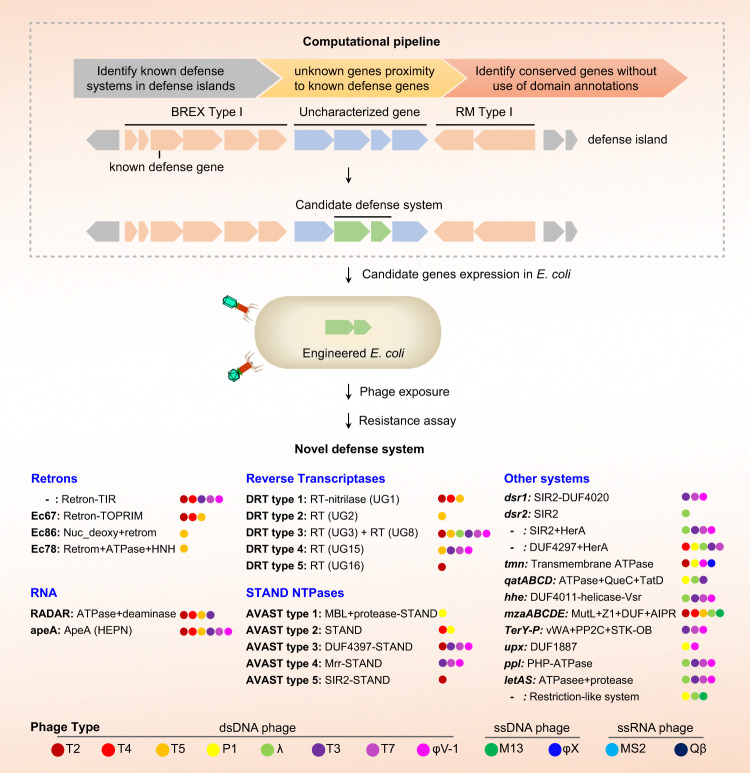
Identifying antiviral defense systems in prokaryotes. Known defense genes in the ‘defense islands’ of bacterial and archaeal genomes act as anchor to screen the neighboring conserved unknown genes, predicted on the basis of the sequences of amino acid and domain-independent annotations, that are then identified as potential novel antiviral systems. The candidates of antiviral systems were cloned into the engineered *E. coli* to investigate whether they can protect from infection by different types of phage. Gao et al. confirmed that twenty-nine defense systems possess antiviral ability. Dot represents the defense system against phage infection: a single-stranded DNA phage (ssDNA phage), double-stranded DNA phage (dsDNA phage), and single-stranded RNA phage (ssRNA phage)

A recent paper in *Science* by Gao et al. extended a comprehensive comparative genomics approach to identify new antiviral systems in prokaryotes for combating invading phages.^1^ These defense systems manifest a variety of host defense mechanisms by utilizing enzymatic activities including reverse transcriptases, adenosine deaminases of RNA editing, and retroms (Fig. 1). Some of these antiviral systems not only enrich our understanding the phage-bacterial interaction but also represent a versatile, powerful tool for biomedical research and biotechnological applications.

Because of the arms race between viruses and prokaryotes, bacteria and archaea have evolved multiple sophisticated antiviral defense strategies to combat phages, for example, restriction modification (RM), abortive infection (Abi) systems, and CRISPR-Cas systems.^2^ Gao et al. described computational analysis of all bacterial and archaeal genomes, encoding over 620 million proteins to discover new antiviral systems. The authors first used known antiviral genes cassettes locating ‘defense islands’ as anchors to search for neighboring uncharacterized genes (Fig. 1), because defense islands often contain abundant antiviral genes that function together between different types of antiviral defense systems with overlap functions to maintain homeostasis. Similar computational pipeline has been published in another *Science* paper published in 2018 by Doron et al. to discover bacterial defense systems from 160 million genes and revealing nine new antiphage systems and one novel anti-plasmid system.^3^ Using this approach, Gao and colleagues detected a total of 7472 putative defense gene families, containing 1687 uncharacteristic function genes, proximity to known defense systems in defense islands.^1^ Further studies identified homologs without use of domain annotations showing that candidate defense gene clusters are evolutionarily conserved across multiple genomes across widespread microorganisms,^1^ indicating the existence of other unidentified defense systems.

To identify these predicted defense gene families, Gao et al. selected 48 candidates from new defense systems to experimentally validate antiviral activities through heterologous reconstitution (Fig. 1). To test multiple variants of candidate defense systems, a collection of one to four homologs of each novel system were chosen to engineer into *Escherichia coli*. A diversity of coliphages were used to infect these engineered *E. coli* strains to investigate their antiviral activity. To the authors’ pleasant surprise, 29 of candidate defense systems (29/48, 60%) provided resistance against phages (Fig. 1), but phage specificity was typically narrow and varied widely across systems.^1^ Of note, whether the other 19 candidate systems contain no antiviral immunity needs future investigation, because they were only tested experimentally under specific laboratory conditions and were expressed in *E. coli* hosts that do not normally express these genes, or lack of appropriate phage targets: defense mechanisms are often effective only against specific phage groups, or inadvertent choice of unfunctional gene sets for testing. It would be interesting to verify that these defense systems truly exert defense functions in the environmental conditions in future.

These validated defense systems demonstrate an abundance ranging from ~0.1 to ~10% of bacterial and archaeal phyla.^1^ One of these, phage restriction by an adenosine deaminase acting on RNA (RADAR), forms three subtypes: RADAR standalone containing only adenosine triphosphatase (ATPase) and adenosine deaminase (*rdrAB*), Csx27-associated RADAR (*rdrABC*), and SLATT-associated RADAR (*rdrABCD*).^1^ Authors suggest that RADAR represents an example of defense via adenosine-to-inosine (A-to-G) RNA editing, in which both RADAR system and phage infection are required for the occurrence of RNA editing.^1^ Broad distribution of editing sites is noted in both phage transcriptomes and engineered *E. coli*, resulting in host growth arrest.^1^ Therefore, RADAR is analogous to editing-dependent Abi, in which prokaryotes commit altruistic cellular suicide to protect larger populations from phage infection. Another interesting system is defense-associated reverse transcriptases (DRTs) identified by enrichment of a family of RTs: DRT type 1 (UG1), DRT type 2 (UG2), DRT type 3 (UG3 and UG8), DRT type 4 (UG15), and DRT type 5 (UG16) (Fig. 1), displaying the distinct pattern of phage resistance.^1^ In DRT type 1, UG1 encodes nitrilase domains. Nitrilases are involved in natural product biosynthesis, including nucleotide metabolism. Authors reveal that nitrilase domain is key for anti-phage ability, exemplifying a non-defense domain that was apparently co-opted for a defense function. DRT type I inhibits late viral gene expression but not early/middle genes and may have no effect on early phage DNA injection.^1^ Another RTs-mediated antiviral defense is dependent on retrons: Retron-TIR (Toll/interleukin-1 receptor domain), Ec67 (Retron-TOPRIM, topoisomerase-primase domain), Ec86 (Nuc_deoxy+retron) and Ec78 (Retron+ATPase+HNH) (Fig. 1).^1^ Retron affects on producing extrachromosomal satellite DNA (msDNA). Gao et al.^1^ find that both synthesis and structure of msDNA are required for defense activity. Retron-TIR systems are associated with TIR domain for sensing pathogen and immune signal transduction, a common feature of innate immune systems in animals, plants, belonging to Thoeris system (*thsAB*).^3^ The motif in Retron-TIR and Thoeris system may be the ancestry of pathogen-associated molecular pattern (PAMP) receptors to recognize pathogens. These data implicate that antiviral defense systems incorporate enzymatic activities against phage infection for recognition and destruction of foreign genetic elements and transcripts.^1^

Furthermore, Gao and colleagues also investigated other defense systems. AVAST systems (antiviral ATPases/NTPases of the STAND superfamily) are associated with nucleoside triphosphatases (NTPases) of STAND (signal transduction ATPases with numerous associated domain) superfamily (Fig. 1).^1^ In eukaryotes, ATPases and GTPases are key components of programmed cell death systems, indicating that they may function through altruistic suicide, similar to Abi. These findings have started to establish long-term development connection between prokaryotic and eukaryotic antiviral defense and programmed cell death mechanism. TerY-phosphorylation triad (TerY-P) system, against T7-like phages, consists of a von Willebrand factor A metal ion binding protein, a serine/threonine protein phosphatase, and a serine/threonine-protein kinase.^1^ Their study hints that TerY-P may control protein phosphorylation, linking kinases to phosphatases. Additional DSR systems possess proteins encoding a SIR2 (sirtutin) deacetylase domain also existing in Thoeris system and prokaryotic Argonaute proteins.^1,3^ ApeA system constitutes HEPN domains, putative ancestors of Cas13 effectors.^1^ qatABCD system includes a four-gene cassette encoding qatA (ATPase), qatB, qatC (QueC), and qatD (TatD)^1^ and QueC participates in small-molecule biosynthesis. Despite similarities in domain architectures among some of novel defense systems, there also exist few other shared features or homologs of known functions, which may present technical hurdles to understand these systems’ functions, and justify further investigation to reveal in-depth mechanisms of phage infection and host protection.

The discovery of hidden stockpile of anti-phage systems is exciting. Gao et al. not only unveil previously uncharacterized multiformity of prokaryotic antiviral defenses from defense islands, but also provide a reminiscence that the virtually unlimited dark matters hidden in the vast majority of microbial genomes are worthy of continued exploration through the approach of computational biology, wet-lab experiments and other novel methods, such as systems biology approaches. Moreover, new discoveries of prokaryotic defenses naturally aroused some excitement about developing tool-kits for molecular biology research, and gene-editing, such as RADAR or ApeA systems for RNA editing. Amazingly, the utility of enzymatic activities of CRISPR-Cas systems for DNA/RNA editing have been transformative.^4,5^ It may be too early to foresee the potential that the novel defense systems discovered by Gao et al., will have innovative technological breakthroughs such as those made by Cas9’s discovery; however, this has ignited tremendous interests in understanding prokaryotes’ marvelous defense systems.
